# TWENTY YEARS OF FOLLOW-UP OF THE MEXICAN HEALTH AND AGING STUDY (MHAS): ITS POTENTIAL FOR CROSS-NATIONAL COMPARISONS

**DOI:** 10.1093/geroni/igae098.2200

**Published:** 2024-12-31

**Authors:** Rebeca Wong

**Affiliations:** The University of Texas Health Sciences Center in San Antonio, San Antonio, Texas, United States

## Abstract

The availability of population-based, national representative, longitudinal data for aging research has been building up for the last twenty years. As a result, the ability to make cross-national research has been enhanced greatly. Almost 20 percent of the publications that use the MHAS data are making cross-national comparisons, but the cross-national work is still in infancy. Most research focuses on documenting health and functionality trajectories and the individual determinants of these trajectories. I will present an overview of what has been done, illustrate with a couple of examples, and conclude with gaps in knowledge that could be supported by cross-national data. The lack of comparable data for many low-middle income countries may be the challenge we can help meet in the next 20 years to advance research on global aging.

